# CO_2_ Methanation: Nickel–Alumina Catalyst Prepared by Solid-State Combustion

**DOI:** 10.3390/ma14226789

**Published:** 2021-11-10

**Authors:** Olga Netskina, Svetlana Mucha, Janna Veselovskaya, Vasily Bolotov, Oxana Komova, Arkady Ishchenko, Olga Bulavchenko, Igor Prosvirin, Alena Pochtar, Vladimir Rogov

**Affiliations:** Boreskov Institute of Catalysis SB RAS, Pr. Akademika Lavrentieva 5, 630090 Novosibirsk, Russia; msa@catalysis.ru (S.M.); jvv@catalysis.ru (J.V.); bolotov@catalysis.ru (V.B.); komova@catalysis.ru (O.K.); arcady.ishchenko@gmail.com (A.I.); obulavchenko@catalysis.ru (O.B.); prosvirin@catalysis.ru (I.P.); po4tar@catalysis.ru (A.P.); rogov@catalysis.ru (V.R.)

**Keywords:** CO_2_ methanation, nickel–alumina catalyst, solid-state combustion method, catalyst activation

## Abstract

The development of solvent-free methods for the synthesis of catalysts is one of the main tasks of green chemistry. A nickel–alumina catalyst for CO_2_ methanation was synthesized by solid-state combustion method using hexakis-(imidazole) nickel (II) nitrate complex. Using X-ray Powder Diffraction (XRD), Transmission electron microscopy (TEM), X-ray photoelectron spectroscopy (XPS), and Hydrogen temperature-programmed reduction (H_2_-TPR), it was shown that the synthesized catalyst is characterized by the localization of easily reduced nickel oxide on alumina surface. This provided low-temperature activation of the catalyst in the reaction mixture containing 4 vol% CO_2_. In addition, the synthesized catalyst had higher activity in low-temperature CO_2_ methanation compared to industrial NIAP-07-01 catalyst, which contained almost three times more hard-to-reduce nickel–aluminum spinel. Thus, the proposed approaches to the synthesis and activation of the catalyst make it possible to simplify the catalyst preparation procedure and to abandon the use of solvents, which must be disposed of later on.

## 1. Introduction

**Figure 1:**
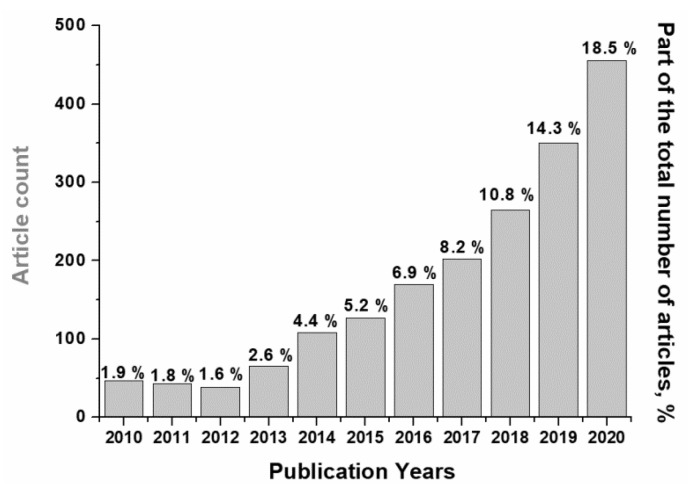
Dynamics of publications on the topic «CO_2_ methanation» over the past 11 years (“Web of Science” database).

In recent years, the scope of publications regarding carbon dioxide (CO_2_) methanation has increased significantly (Figure 1), which is due to growing interest in processes of CO_2_ utilization and hydrogen accumulation for the production of synthetic natural gas [1,2,3].

In addition, this process is considered as a promising approach to enhance the concentration of methane within biogas as containing up to 50 vol% of CO_2_ [4,5]. Currently, there are already technological solutions being implemented in the industry [6], but the maximum possible methane yield has not been achieved yet as for laboratory conditions.

From a thermodynamic point of view, CO_2_ methanation is a favorable and highly exothermic process, but a reversible one.

CO_2_ + 4H_2_ ↔ CH_4_ + 2H_2_O    ΔG_298_ = −114 kJ mol^−1^; ΔH_298_ = −165 kJ mol^−1^ (1)

Theoretical calculations have revealed that a high CO_2_ conversion may be achieved at a temperature below 300 °C, and, upon pressure increase, it may be enhanced [7]. However, within this temperature range, the rate of eight-electron reduction of CO_2_ to CH_4_ (Figure S1) is low due to high kinetics barriers [8]. To increase effective CO_2_ conversion, especially at atmospheric pressure within the low-temperature region (150–300 °C), catalysts are required. Their active component is limited to metals of group VIII [9,10,11,12]. Herewith, the preference is given to nickel, which combines successfully low cost with acceptable catalytic activity in CO_2_ methanation [13,14,15]. Various metal oxides are used as a support for nickel [16], but most publications consider catalysts based on cheap and commercially available γ-alumina with a mesoporous structure which is changed moderately up to 600 °C compared to ZrO_2_ and CeO_2_ [17]. The high thermal stability of nickel–alumina catalysts is a very important parameter since the exothermic nature of CO_2_ methanation may lead to a reaction layer strong heating within large-scale reactors.

The mechanism of CO_2_ methanation over nickel–alumina catalysts is still considered to be a debatable issue [18,19], but its implementation requires the availability of metallic nickel necessarily [20]. As shown in [21], the higher is the Ni^0^/Ni^2+^ ratio, the more effective is the catalyst. It is believed that the reduced metal activates both hydrogen [22] and carbon dioxide. CO_2_ activation is carried out through the formation of CO as an intermediate, according to the XPS data [23]. It is also determined by the density functional method that the most energetically favorable way of CO_2_ methanation on the (111) plane of nickel is through CO [24]. It should be noted that the stage of CO_2_ dissociation limits the reaction rate [25]. Moreover, despite hydrogen adsorption, the surface of nickel particles is partially oxidized according to Inelastic Neutron Scattering data [26].

Alumina remains practically an inert material for the activation for the reagents up to 300 °C [27] since carbon dioxide is adsorbed irreversibly upon its strong basic centers within this temperature range [22,28]. The resulting carbonate species decompose with CO_2_ desorption only at temperatures above 500 °C [29]. In this regard, the role of aluminum oxide in low-temperature methanation of CO_2_ (150–300 °C) is to ensure high dispersion of the active component and increase the strength and thermal stability of the catalyst, including the preservation of the porous structure during local overheating in the catalyst bed.

Nickel oxide is preferred as a precursor of the active component of nickel–alumina catalysts for CO_2_ methanation since it is reduced by hydrogen under milder conditions than nickel–aluminum spinel [30]. Traditionally, industrial catalysts are reduced by a nitrogen–hydrogen mixture without carbon oxides because they may cause active component loss due to the formation of nickel carbonyl with a boiling point of about 42 °C [31]. However, from a practical point of view, it is advisable to reduce a catalyst directly by a reaction mixture (H_2_/CO_2_) at a temperature ≤350 °C since within this temperature range production of methane with high selectivity is advantageous thermodynamically [7].

It was noted that the CO_2_ methanation rate is influenced not only by the nature of the active component and the support [32,33] but also by their synthesis conditions [34,35]. Industrial nickel–alumina catalysts are obtained by co-precipitation followed by high-temperature treatment to form nickel oxide and alumina [36]. Now nickel precursor of the active component is supported on the surface of commercial alumina using different approaches: wet impregnation [37], evaporation induced self-assembly technique [38], chemical vapor deposition [39], a surfactant-free sol-gel technique [40], mechanochemical activation [41], microwave-assisted hydrothermal synthesis [42], and ultrasound-assisted co-precipitation method [43]. The preparation methods of CO_2_ methanation catalysts, as a rule, include two high-temperature stages (500–700 °C): (I) nickel oxide formation and (II) its reduction by hydrogen up to the active state. In addition, most of the above catalyst preparation methods involve the use of solvents, which must then be disposed of. Therefore, the development of energy-saving solvent-free methods for the synthesis of nickel–alumina CO_2_ methanation catalysts remains an urgent challenge.

At the moment, a new direction of nanomaterials preparation by combustion method is being actively developed [44]. Due to the rapid redox reactions, the released heat is localized within a finite layer, establishing a high-temperature zone where metal-containing nanoparticles are formed. They are well-crystallized and can be used as catalysts in important chemical processes including CO_2_ methanation. Currently, aqueous solutions of nickel nitrate with urea and aluminum hydroxide [34] or nitrate [45] are used for the synthesis of effective nickel-based catalysts by solution combustion technique and self-propagating high-temperature synthesis [46].

This work aims to develop a new approach to the synthesis of nanoscale nickel–alumina catalysts for CO_2_ methanation based on solid-state combustion (SSC) without using any solution [44]. Organometallic compounds prepared by solvent-free dry-melt synthesis [47,48] were used as Ni catalyst precursors. In addition to metals, their composition includes energy-intensive organic ligands and oxygen-containing anions, ensuring the initiation of the combustion process during short-term thermal exposure [49,50]. We prepare nickel–alumina catalyst by solid-state combustion from a solid mixture of γ-Al_2_O_3_ and a hexakis-(imidazole) nickel (II) nitrate complex. Since there are no standardized conditions to research CO_2_ methanation, in this work the synthesized catalyst was compared with industrial NIAP-07-01 catalyst [36] obtained by co-precipitation. This allows making a correct conclusion about the prospects of the SSC technique for the synthesis of effective catalysts to ensure low-temperature CO_2_ methanation.

## 2. Materials and Methods

### 2.1. Synthesis of Hexakis-(Imidazole) Nickel (II) Nitrate Complex

Hexakis-(imidazole) nickel (II) nitrate complex was synthesized from imidazole and nickel nitrate without using solvents. The synthesis was performed within a ceramic crucible at 150 °C. When nickel nitrate (Ni(NO_3_)_2_·6H_2_O, CAS No 13478-00-7) was added in a colorless imidazole (C_3_H_4_N_2_, CAS No 288-32-4) a reaction mixture was colored in green, and then crystallized instantly as a blue powder. The obtained sample was cooled at room temperature and separated into fractions. Nickel–alumina catalyst was prepared using a solid complex with particle size below 0.04 mm. 

Oxygen balance of complex was calculated using the following equation:

(2) $$\mathrm{Oxygen}\;\mathrm{balance}\;(\%)\;=\;\frac{-1600}{\mathrm{Molar}\;\mathrm{mass}}\cdot (2C+\frac{H}{2}-\mathrm{MO})$$

where C is the quantity of carbon atoms, H is quantity of hydrogen atoms, O is the quantity of oxygen atoms, and M is the quantity of metal atoms.

### 2.2. Catalyst Preparation

The γ-Al_2_O_3_ (SASOL Pural TM 100; BET specific surface area is 158 m^2^·g^−1^) supported nickel catalyst was prepared by solid-state combustion technique. Alumina was ground to a fraction of below 0.04 mm and calcined at 500 °C per 4 h. Then a mechanical mixture of alumina (4.00 g) with hexakis-(imidazole) nickel (II) nitrate complex (10 g) was placed into a ceramic crucible which was preheated up to 300 °C. Melting of the complex was observed and then it ignited with the release of a large amount of gas. It is worth noting that the nickel–alumina catalyst prepared by solid-state combustion technique was not calcined. The obtained powder was granulated. A fraction with a particle size of 0.25–0.5 mm was tested in the CO_2_ methanation. Nickel–alumina catalyst was designated as Ni-Al_2_O_3_-SSC. 

Industrial NIAP-07-01 catalyst (TU 2178-003-00209510-2006, NIAP-CATALYST Ltd., Novomoskovsk, Russia) was prepared by precipitation of nickel hydroxide and loaded over γ-Al_2_O_3_ in one step [36]. Excess water was removed by drying at 120 °C and then the sample was calcined at a temperature of above 400 °C [51]. For comparative studies, a catalyst with a particle size between 0.25 and 0.5 mm was used.

### 2.3. Catalyst Characterization

Inductively coupled plasma atomic emission spectrometry (Optima 4300 DV, PerkinElmer, Waltham, MA, USA) was used to determine Ni and Al in complex and catalysts. Using an automatic CHNS analyzer EURO EA 3000 (Euro Vector S.p.A., Castellanza, Italy) the contents of C, H, and N were measured. The determination error is in the range of ±0.3–0.5 wt%. From the obtained data, the composition of the complex was calculated and compared with the theoretical ones (Table S1).

The textural characteristics of the catalysts were determined by low-temperature nitrogen adsorption/desorption. Using an ASAP-2400 (Micromeritics Instrument Corporation, Norcross, GA, USA) the adsorption isotherms were obtained at 77 K. The relative error of determination was ±6%.

ATR FTIR spectra were recorded on an Agilent Cary 600 (Agilent Technologies, Santa Clara, CA, USA) spectrometer equipped with a Gladi ATR attachment (PIKE Technologies, Madison, WI, USA).

For thermal analysis a Netzsch STA 449 C Jupiter instrument (NETZSCH, Selb, Germany) equipped with a DSC/TG holder was used. The measurements were performed in the temperature range of 30–600 °C under a He flow (30 mL·min^−1^). The heating rate: 5 °C·min^−1^ for complex; 10 °C·min^−1^ for catalysts. The sample mass was 4 mg.

Using a D8 Advance diffractometer (Bruker AXS GmbH, Karlsruhe, Germany) equipped with a Lynxeye linear detector (CuK_α_ radiation, λ = 1.5418 Å) the phase composition of complex and catalysts were characterized. The lattice parameter of γ-Al_2_O_3_ was determined from symmetric reflection at [440] (2θ = 66.9°). The standard deviation was ±0.001 Å.

The structure and microstructure of samples were surveyed by ThemisZ transmission electron microscope with a limiting resolution of 0.07 nm (Thermo Fisher Scientific, Waltham, MA, USA). Using energy dispersive spectrometer SuperX Thermo Fisher Scientific (HAADF) the elemental maps were obtained. Samples for research were fixed onto standard copper grids using ultrasonic dispersion in anhydrate ethanol.

A SPECS photoelectron spectrometer with a PHOIBOS-150-MCD-9 hemispheric analyzer and a FOCUS-500 monochromator (SPECS Surface Nano Analysis GmbH, Berlin, Germany) (AlK_α_ radiation, 150 W, hν = 1486.74 eV) were used to record XPS spectra. The binding energy (BE) scale of the spectrometer was pre-calibrated using Au4f_7/2_ (84.0 eV) and Cu2p_3/2_ (932.6 eV) core-level peaks. The binding energies were determined with an accuracy of ±0.1 eV. The XPS analysis depth was about 5 nm. The samples were supported on a conducting scotch. The sample charge was taken into account using C1s lines (284.8 eV). Analysis of individual spectra of elements allowed to determine their electronic state and to calculate the ratio of oxidized to reduced nickel onto catalyst surface, regarding element sensitivity coefficients [52].

H_2_-TPR experiments were realized in a flow reactor with gradient-free furnace. The temperature was changed from 30 to 900 °C at a heating rate of 10 °C·min^−1^. For uniform heating, a quartz glass (fraction: 0.4–0.7 mm) was added to the sample. The hydrogen flow rate (10% H_2_ in argon) was 40 mL·min^−1^. A thermal conductivity detector was used to determine the hydrogen content in the outlet gas flow. A trap with liquid nitrogen was used to remove impurities.

### 2.4. Experimental Setup

All the catalytic experiments were performed in a fixed-bed reactor with an inner diameter of 12.8 mm under atmospheric pressure. 318 mg of catalyst was placed into the reactor, and heated using a tubular furnace. The temperature was controlled by a thermocouple installed in a catalyst bed. Catalytic tests were carried out at various temperatures from 150 to 450 °C with a step of 50 °C. Typically, the catalyst was kept in the reaction mixture for 20 min at each temperature. In the case of activation, the duration of the isothermal step was varied.

The mixture H_2_, CO_2,_ and Ar flow with the volume ratio of 16:4:80 was introduced into the catalyst bed with a gas mass space velocity of 19,000 mL/g_cat_·h. The flow rates were controlled by RRG-12-36 flow meter (“Eltochpribor”, Moscow, Russia) with an accuracy of 1%. The gas composition at the reactor’s outlet was determined using Agilent Cary 600 FTIR-spectrometer (Agilent Technologies Australia, Melbourne, Australia). The internal volume of the gas cell is 100 cm^3^ and the optical path length was 10 cm.

The amount of carbon-containing products in the reaction medium before and after the reactor was determined using nondispersive infrared sensors (Dynament Limited, Mansfield, UK). The obtained data were used to calculate CO_2_ (*X_CO_*_2_) conversion as follows:

(3) $$X_{CO_{2}}=\left(1-\frac{C_{out}}{C_{in}}\cdot \frac{U_{out}}{U_{in}}\right)\cdot 100\%$$

where *C_in_* and *C_out_* are CO_2_ concentrations at the inlet and outlet of the reactor, and *U_in_* and *U_out_* are gas flow rates at the inlet and outlet of the reactor. Taking into account reaction stoichiometry and the high content of argon in the reaction medium, the *U_in_/U_out_* ratio was in the range from 0.92 to 1.

## 3. Results and Discussion

### 3.1. Study of the Organometallic Precursor of the Nickel–Alumina Catalyst

An organometallic precursor of nickel–alumina catalyst was prepared first by melting nickel nitrate and imidazole at 150 °C. As a result, a solid powder of blue color was obtained. Its composition corresponded to the calculated composition of the hexakis-(imidazole) nickel (II) nitrate (Table S1).

**Figure 2:**
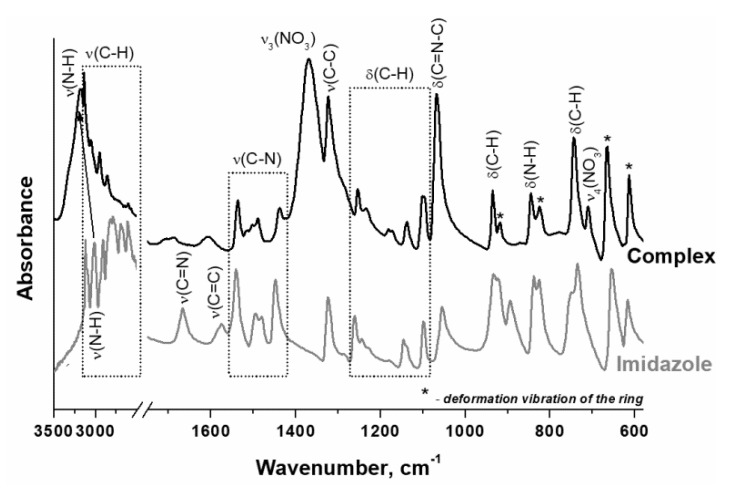
ATR FTIR spectra of the prepared hexakis-(imidazole) nickel (II) nitrate complex and imidazole.

It should be noted that imidazole is an amphoteric compound since it contains a weak acid group (>N–H) in a five-membered heterocycle, and a nitrogen atom (–N=) containing lone pair available for bonding to proton. Thus, the crystal structure of imidazole is stabilized by intermolecular hydrogen bonds [53], which are absent in complex compounds. In complex compounds, the lone pair of the pyridine nitrogen atom is coordinated by the metal cation. In this case, the N–H group becomes isolated, and the interatomic distance is shortened. According to ATR FTIR spectroscopy data, this leads to an increase in the intensity of stretching vibrations of the N–H bond and a shift of its absorption band (a.b.) to the high-frequency region (Figure 2). It was found that the ATR FTIR spectra of the prepared complex and imidazole (Figure 2) have a very similar set of a.b., which indicates the retention of the imidazole structure during the synthesis of the complex by melting of imidazole and nickel nitrate.

**Figure 3:**
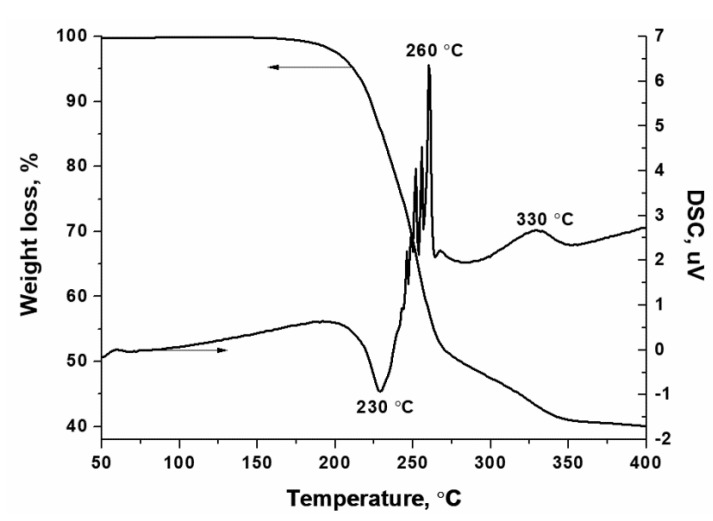
Thermal decomposition of the synthesized hexakis-(imidazole) nickel (II) nitrate complex (helium, 5 °C·min^−1^).

Additional evidence for the formation of hexakis-(imidazole) nickel (II) nitrate complex is provided by thermal analysis (Figure 3) and XRD (Figure S2) data. Thus, the DTA curves show no endothermic peaks at 90 and 265 °C, which corresponds to the melting and boiling of imidazole, respectively. Moreover, there is no mass loss and thermal effects due to the decomposition of nickel nitrate hexahydrate [54]:

Ni(NO_3_)_2_·6H_2_O → Ni(NO_3_)_2_·4H_2_O + 2H_2_O                43 °C  Δm = 12.3 wt% (4)

Ni(NO_3_)_2_·4H_2_O → Ni(NO_3_)_2_·2H_2_O + 2H_2_O              80 °C  Δm = 24.8 wt% (5)

Ni(NO_3_)_2_·2H_2_O → Ni(NO_3_)(OH)_2_·H_2_O + NO_2_          145 °C  Δm = 40.6 wt% (6)

Ni(NO_3_)(OH)_2_·H_2_O → Ni(NO_3_)(OH)_1.5_O_0.25_·H_2_O + 0.25H_2_O  190 °C  Δm = 42.0 wt% (7)

Ni(NO_3_)(OH)_1.5_O_0.25_·H_2_O → 0.5Ni_2_O_3_ + HNO_3_ + 1.25H_2_O   250 °C  Δm = 71.8 wt% (8)

3Ni_2_O_3_ → 2Ni_3_O_4_ + 0.5O_2_                  250 °C  Δm = 72.2 wt% (9)

Ni_3_O_4_ → 3NiO + 0.5O_2_                  300 °C  Δm = 74.0 wt% (10)

According to thermal analysis (Figure 3), a noticeable decomposition of the complex begins at temperatures above 150 °C with an endothermic effect, but the main weight loss above 230 °C takes place with the release of a large amount of heat. The saw-like form of the exothermic peak indicates the uneven release of heat, which is probably due to the stepwise ligand removal with their simultaneous oxidation. However, complete oxidation of the organic ligand does not occur because their weight loss was only 50% at 270 °C, but not as expected (up to 87.3%). Therefore, the exothermic peak at 330 °C can be attributed to the oxidation of undecomposed complex with the release of gaseous products. For example, in this temperature range, nickel oxide interacts with carbon formed as a result of the carbonization of imidazole.

NiO + C → Ni + CO           300–400 °C
(11)

In general, summing up the study, we can draw the following conclusion. The blue powder obtained by melting nickel nitrate and imidazole at 150 °C is a hexakis-(imidazole) nickel (II) nitrate complex, in which the nickel cation is coordinated by imidazole molecules through the lone pair of the nitrogen atom. The simultaneous presence of imidazole as an energy-intensive ligand and a nitrate-anion as an oxidizing agent makes it possible to use this complex for the synthesis of the nickel oxide by the SSC method.

### 3.2. Study of Nickel–Alumina Catalyst

Synthesized hexakis-(imidazole) nickel (II) nitrate complex was used as an organometallic precursor of a nickel–alumina catalyst (Ni-Al_2_O_3_-SSC) for CO_2_ methanation. The formation of nickel oxide on the alumina surface was carried out by the SSC method. The prepared Ni-Al_2_O_3_-SSC catalyst was studied in comparison with the industrial NIAP-07-01 catalyst of CO_2_ methanation, which was prepared by wet precipitation [36].

#### 3.2.1. Catalyst Composition

For the Ni-Al_2_O_3_-SSC catalyst, the ratio of the initial components was determined based on the final content of nickel oxide not less than 20 wt%. As a result, the catalyst was prepared with a calculated NiO content of 20.8 wt% (Table 1). It should be noted that in the industrial NIAP-07-01 catalyst a nickel content was 1.5 times higher (31.3 wt% in terms of oxide) than that the Ni-Al_2_O_3_-SSC catalyst prepared by the SSC method.

**Figure 4:**
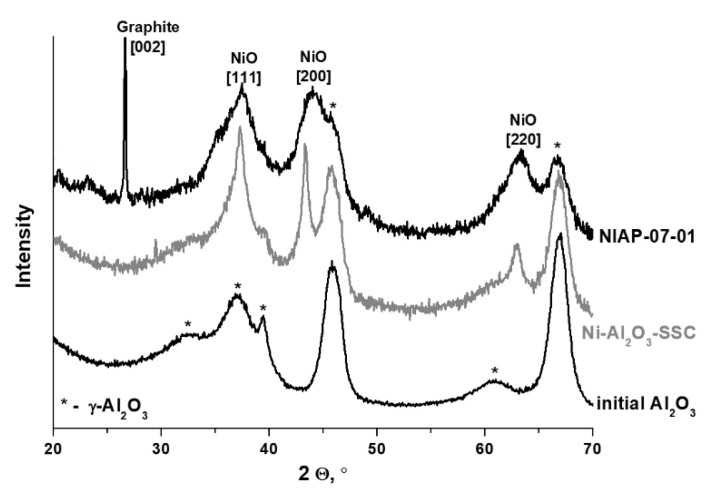
XRD patterns of the NIAP-07-01 and Ni-Al_2_O_3_-SSC catalysts.

**Figure 5:**
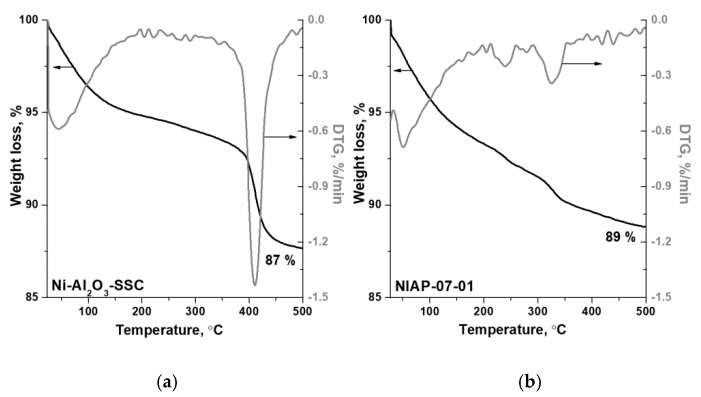
Thermal analysis data for (**a**) Ni-Al_2_O_3_-SSC and (**b**) NIAP-07-01 catalysts (helium, 10 °C·min^−1^).

In the Ni-Al_2_O_3_-SSC and NIAP-07-01 catalysts, an aluminum percentage is about 50 ± 6 wt%, which is lower than expected if metal was present as alumina only, according to XRD data (Figure 4). Impurities can be the reason for the low aluminum content. According to FTIR spectroscopy data, when the catalysts are heated in an inert atmosphere (argon) carbon dioxide, water and ammonia are released (Figure S3). Therefore, the catalysts contain carbonates, water, and products of incomplete oxidation of imidazole as impurities. According to thermal analysis (Figure 5), their content is 13 and 11 wt% for Ni-Al_2_O_3_-SSC and NIAP-07-01, respectively.

Besides, the low aluminum content (Table 1) can be associated with phase inhomogeneity of catalysts, especially for an industrial NIAP-07-01 catalyst. Its XRD pattern contains an intense peak of graphite (at angle 26.5°), as well as weak intensity peaks at low angles (2θ = 20.4, 23.1°), which could not be interpreted unambiguously. Moreover, for NIAP-07-01, there is an increase of spinel cell parameter of alumina (Table 2), probably due to the intercalation of nickel cations with a larger ionic radius than Al^3+^.

In the case of the Ni-Al_2_O_3_-SSC catalyst, the spinel cell parameter changes insignificantly (Table 2). Apparently, under conditions of short-term high-temperature heating (~1000 °C), nickel oxide is rapidly formed from the hexakis-(imidazole) nickel (II) nitrate complex. Thus, the probability of the intercalation of nickel ions into the alumina lattice decreases.

#### 3.2.2. Local Analysis of Catalyst Particles

**Figure 6:**
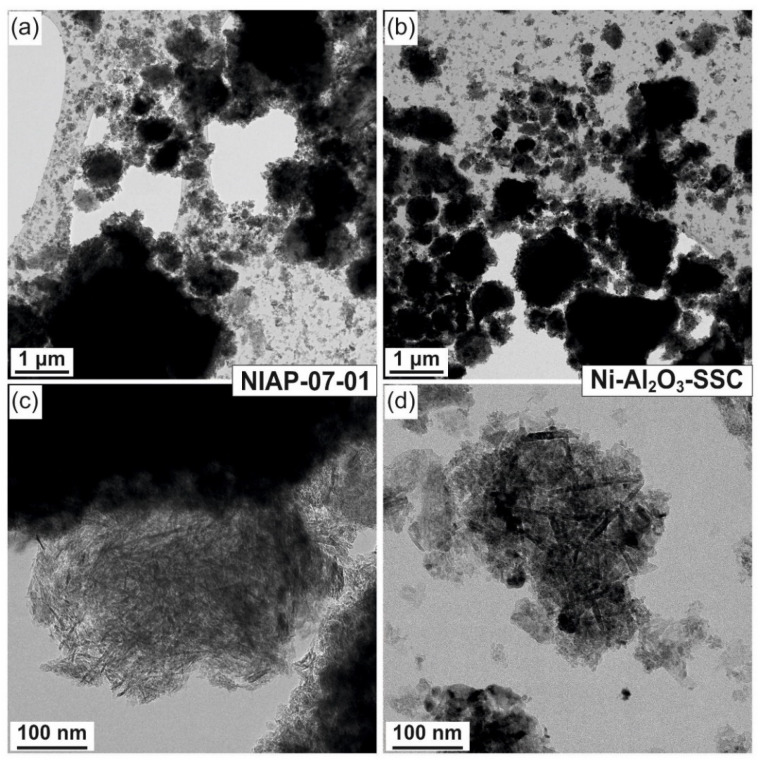
Morphology of (**a**) NIAP-07-01 and (**b**) Ni-Al_2_O_3_-SSC catalysts, as well as individual particles of (**c**) NIAP-07-01 and (**d**) Ni-Al_2_O_3_-SSC catalysts.

**Figure 7:**
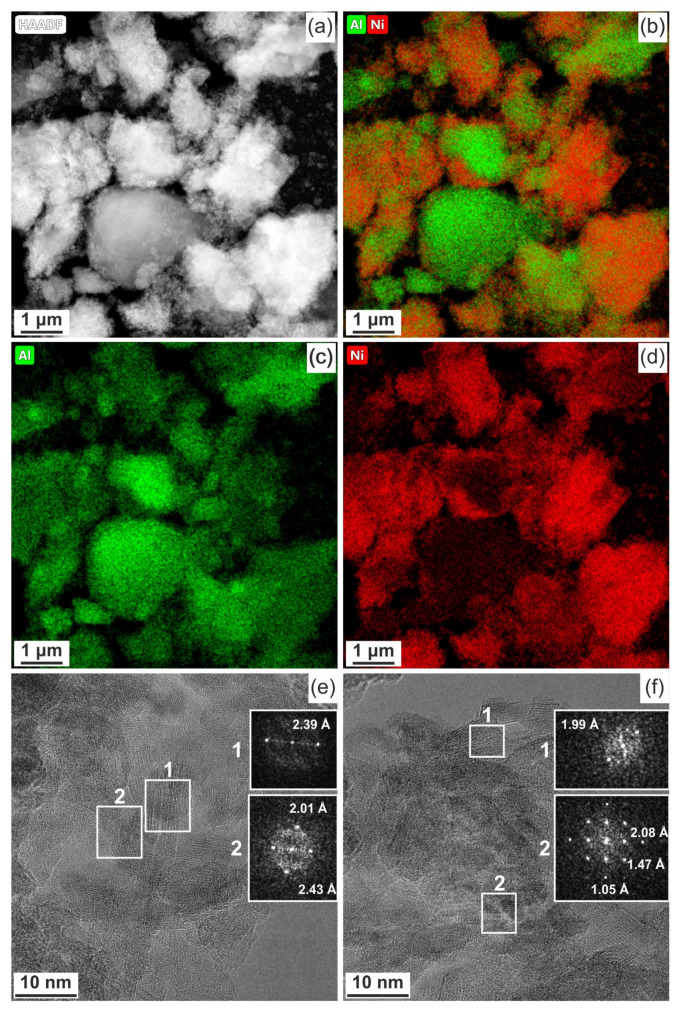
NIAP-07-01 catalyst: (**a**–**d**) compositional elemental mapping and (**e**,**f**) fast Fourier transform (FFT) patterns are calculated from the TEM image of individual particles.

**Figure 8:**
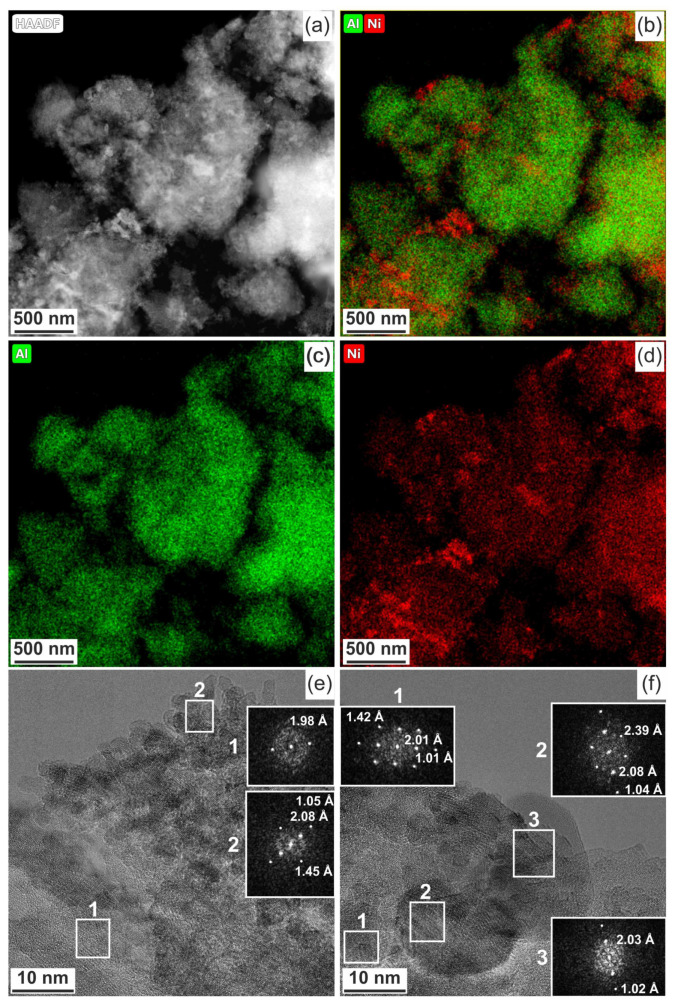
Ni-Al_2_O_3_-SSC catalyst: (**a**–**d**) compositional elemental mapping and (**e**,**f**) fast Fourier transform (FFT) patterns are calculated from the TEM image of individual particles.

Morphology and composition of Ni-Al_2_O_3_-SSC and NIAP-07-01 particles catalysts were studied by HR TEM. It was noted that the particles of catalysts are of similar size. It varies from a few microns to 10s of nanometers (Figure 6a or Figure 6c). They are loose aggregates from thin plate-like nanoparticles (Figure 6b or Figure 6d). Elemental mapping indicates a different distribution of aluminum and nickel in particles of studied catalysts (Figure 7 and Figure 8). Most of the industrial NIAP-07-01 catalyst particles have an almost uniform distribution of nickel in the aluminum-containing particles, but there are also aluminum-containing particles decorated with a nickel phase (Figure 7b–d). The analysis of individual particles showed that they had crystal faces with interplanar distances of 2.39, 1.99, 2.01, 2.43, 2.08, 1.47, and 1.05 Å (Figure 7e,f). These values almost coincide with the similar characteristics for alumina γ-Al_2_O_3_ (PDF 10-425: [311]—2.390 Å, and [400]—1.977 Å), nickel–aluminum spinel NiAl_2_O_4_ (PDF 10-339: [400]—2.013 Å, [311]—2.427 Å, and [800] 1.006 Å) and nickel oxide NiO (PDF 47-1049: [200]—2.089 Å, [220]—1.477 Å, and [400]—1.044 Å). By comparing these results with the XRD pattern (Figure 4), it can be concluded that an increase in the lattice parameters of alumina is due to the presence of the nickel–aluminum spinel in the industrial NIAP-07-01 catalyst.

When the Ni-Al_2_O_3_-SSC catalyst was studied in detail (Figure 8), it was shown that its particles predominantly contain alumina (interplanar distance is 1.98 Å). If Figure 8a,b are compared, it can be seen that nickel is mainly concentrated on the surface of the alumina particles. The nickel-containing phase consists of a mixture of nickel oxide (2.08, 1.45, and 1.05 Å) and nickel–aluminum spinel (2.01, 1.42, and 1.01 Å), according to their interplanar determined by FFT patterns (insets in Figure 8e,f). Furthermore, the Ni-Al_2_O_3_-SSC catalyst is characterized by the presence of spherical particles with interplanar spacings of 2.03 and 1.02 Å (Figure 8f), which corresponds to metallic nickel (PDF 4-850: [111]—2.034 Å, and [222]—1.017 Å).

Thus, the formation of the phase of nickel oxide and its anchorage on the alumina surface occurs by the SSC method using a hexakis-(imidazole) nickel (II) nitrate complex. In addition, a small part of the nickel is reduced, probably due to the interaction of nickel oxide with carbon (Equation (11)) formed in the deficiency of an oxidizing agent, since the organometallic precursor has a strongly negative oxygen balance (Table S1).

#### 3.2.3. State of Nickel on the Catalyst Surface

**Figure 9:**
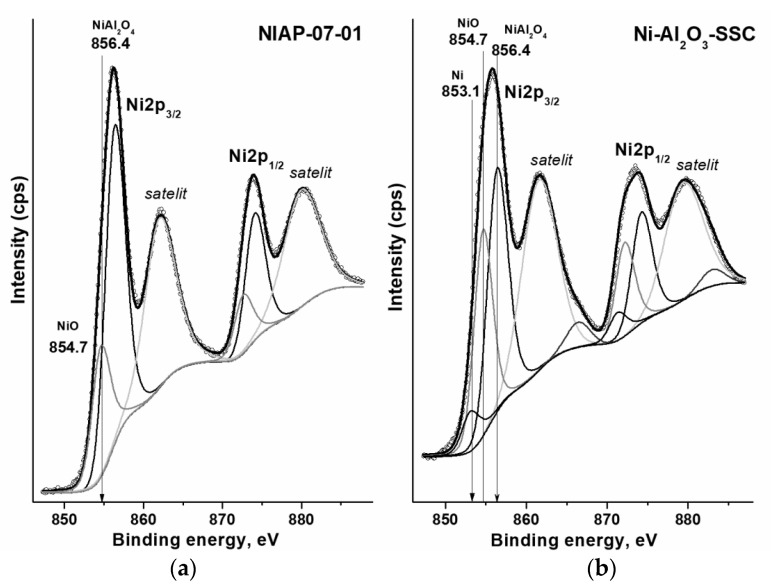
Deconvoluted high-resolution XPS spectra of Ni2p for (**a**) NIAP-07-01 and (**b**) Ni-Al_2_O_3_-SSC catalysts.

The type of compounds on the surface of catalyst particles affects the formation of the active component. Each compound has its own set of electronic states of elements. By the XPS method (Figure 9), the electronic state of nickel on the surface of Ni-Al_2_O_3_-SSC and NIAP-07-01 catalysts was studied. From XPS spectra it is seen that the Ni2p_3/2_ lines are not symmetric and their deconvolution allows the identification of several electronic states of the metal. The lowest value of the binding energy of 853.1 eV corresponds to metallic nickel Ni^0^ [55] while the binding energies of 854.7 and 856.4 eV are characteristic of nickel oxide NiO [56] and nickel–aluminum spinel with composition NiAl_2_O_4_ [57], respectively. At the same time, the industrial NIAP-07-01 catalyst is characterized by only two electronic states with binding energies (Figure 9a) corresponding to nickel oxide and nickel–aluminum spinel. Since the atom ratio of NiAl_2_O_4_/NiO is 4.76, nickel–aluminum spinel predominates on the surface of the industrial NIAP-07-01 catalyst (Table S2). The same trend is typical for Ni-Al_2_O_3_-SSC catalyst (Figure 9b), but the nickel–aluminum spinel content is only 1.54 times that of nickel oxide. It should be noted that a small amount of reduced nickel (853.1 eV) is present on the surface of this catalyst. This is consistent with the HR TEM results (Figure 8f).

**Figure 10:**
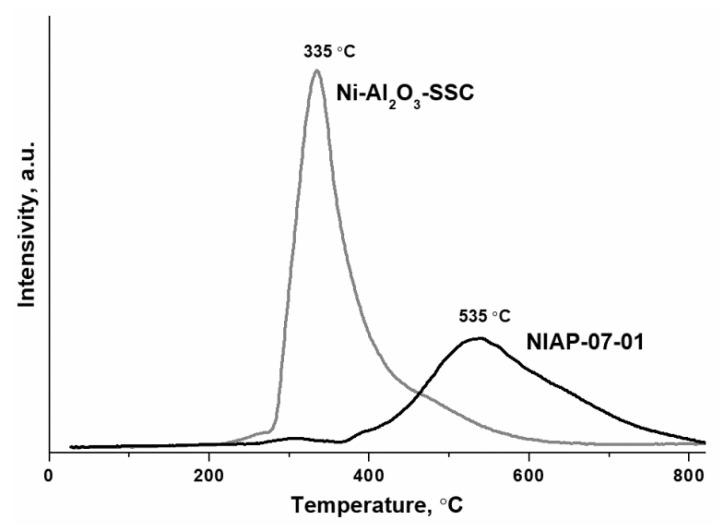
H_2_-TPR profiles of NIAP-07-01 and Ni-Al_2_O_3_-SSC catalysts.

Thus, the developed SSC method using a hexakis-(imidazole) nickel (II) nitrate complex makes it possible to synthesize the nickel–alumina catalyst with a higher content of nickel oxide on the alumina surface as compared to the industrial NIAP-07-01 catalyst of CO_2_ methanation. The surface placement of nickel oxide should favorably influence the formation of the active component since it provides direct access for hydrogen to nickel oxide as a catalyst precursor, which is reduced at a lower temperature than nickel–aluminum spinel [30]. Thus, the reduction of bulk NiO takes place up to 300 °C [58], the reduction of NiO species that interact weakly with the alumina is at 300–400 °C, the reduction of NiO species with moderately strong support interactions is at 400–500 °C, while the reduction of NiAl_2_O_4_ is observed at temperatures above 600 °C [59,60]. Indeed, the H_2_-TPR method (Figure 10) confirmed that the Ni-Al_2_O_3_-SSC catalyst begins to reduce already at 250 °C, and the industrial NIAP-07-01 catalyst is reduced after reaching 300 °C.

Ni-Al_2_O_3_-SSC catalyst consumes more hydrogen compared to NIAP-07-01. This is a good precondition for the formation of a more active catalyst due to the larger amount of reduced nickel in the Ni-Al_2_O_3_-SSC catalyst, despite lower Ni content than with the industrial NIAP-07-01 catalyst (Table 1). However, in the synthesis of the catalyst by the SSC method the formation of impurities cannot be prevented. Survey XPS spectrum confirms the presence of carbon and nitrogen in Ni-Al_2_O_3_-SSC catalyst (Figure S4). This explains the release of carbon dioxide, water, and ammonia during the thermal treatment of this catalyst (Figure S3).

#### 3.2.4. Porous Structure of Catalysts

For the kinetics of catalytic reactions, internal diffusion is an important characteristic. The diffusion rate depends on the porous structure of the catalyst. Textural characteristics of Ni-Al_2_O_3_-SSC and NIAP-07-01 catalysts were studied by low-temperature nitrogen adsorption/desorption. The obtained isotherms (Figure S5) correspond to type IV according to the IUPAC classification [61].

**Figure 11:**
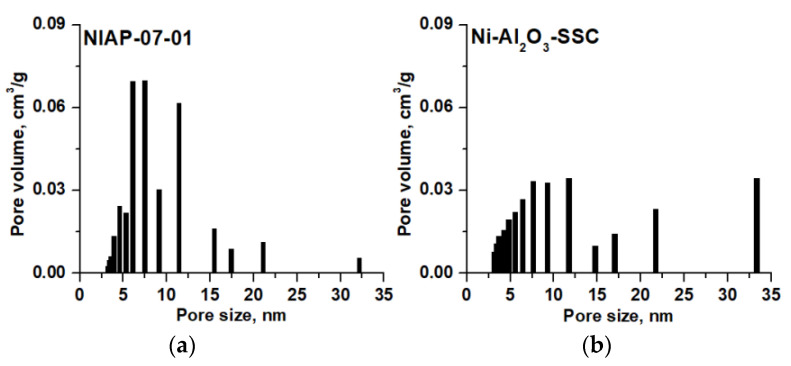
Pore size distribution for (**a**) NIAP-07-01 and (**b**) Ni-Al_2_O_3_-SSC catalysts.

The analysis of the data obtained showed that for Ni-Al_2_O_3_-SSC and NIAP-07-01 catalysts the pore size distribution is different (Figure 11), but the total pore volume has similar values (Table 3). Consequently, the effect of the internal diffusion of reactants on the CO_2_ methanation rate will be similar for these catalysts.

### 3.3. Study of CO_2_ Methanation

#### 3.3.1. Catalyst Activation

The catalytic conversion of CO_2_ to CH_4_ requires nickel metal particles located on the surface of the nickel–aluminum catalysts. Traditionally, the reduction of nickel–alumina catalyst is carried out in a flow of pure hydrogen [29] or inert gas diluted hydrogen [62] at temperatures from 500 to 700 °C. As noted earlier (Figure 10), a high temperature is required to form the active component of catalyst from nickel–aluminum spinel.

NiO + H_2_ → Ni + H_2_O           200–400 °C (12)

NiAl_2_O_4_ + H_2_ → Ni + H_2_O + Al_2_O_3_      500–800 °C (13)

In addition, the high-temperature reduction of the catalyst makes it possible to prevent the loss of the active component due to the possible formation of quickly evaporating nickel carbonyl at temperatures below 140 °C, as well as the formation of nickel carbide at 270 °C. However, these processes are observed only in the presence of carbon monoxide.

Ni + 4CO → Ni(CO)_4_             50–140 °C (14)

3Ni + 2CO → Ni_3_C + CO_2_            500–800 °C (15)

To simplify the procedure for the reduction of CO_2_ methanation catalysts, the activation of Ni-Al_2_O_3_-SSC and NIAP-07-01 catalysts in a flow of reaction mixture (H_2_:CO_2_:Ar = 16:4:80) was studied. Since CO_2_ and CH_4_ have characteristic absorption bands [63] a qualitative assessment of the nickel reduction with varying the temperature of activation was performed by FTIR spectroscopy.

**Figure 12:**
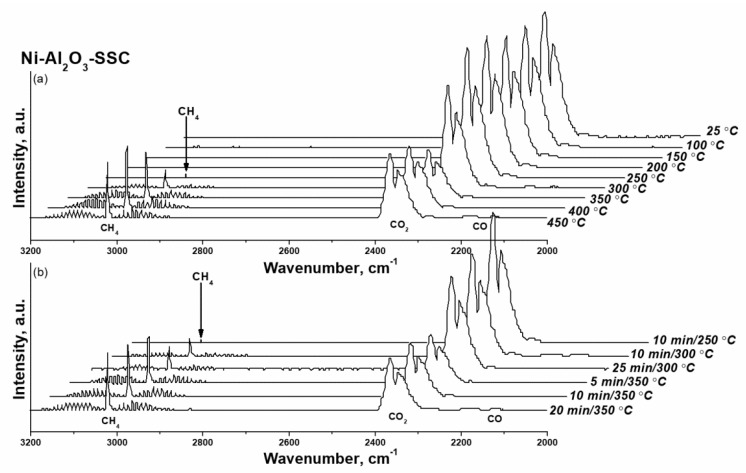
FTIR spectra of the gas at the outlet of the reactor when the Ni-Al_2_O_3_-SSC catalyst was activated at varying (**a**) the temperature and (**b**) duration of the process. Activation conditions: volume ratio is H_2_:CO_2_:Ar = 16:4:80, and flow rate is 19,000 cm^3^·g_cat_**^−^**^1^·h**^−^**^1^.

**Figure 13:**
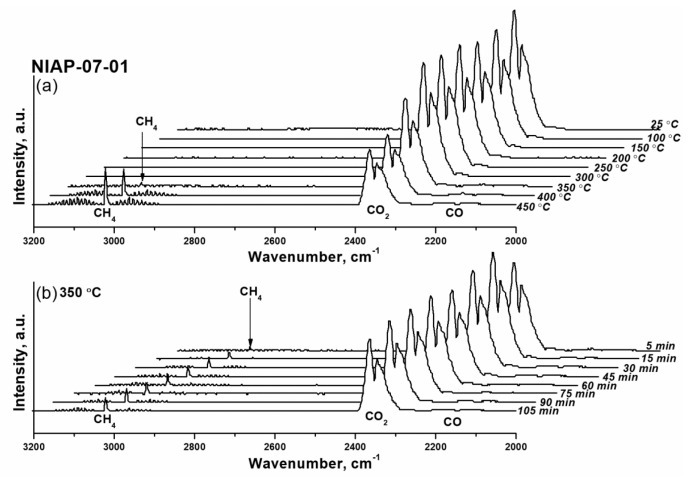
FTIR spectra of the gas at the outlet of the reactor when the industrial NIAP-07-01 catalyst was activated at varying (**a**) the temperature and (**b**) duration of the process. Activation conditions: volume ratio is H_2_:CO_2_:Ar = 16:4:80, and flow rate is 19,000 cm^3^·g_cat_**^−^**^1^·h**^−^**^1^.

As shown in Figure 12, for Ni-Al_2_O_3_-SSC catalyst distinguishable a.b. of methane are already observed at 250 °C. Within an hour, their intensity does not change, which is due to the small amount of reduced nickel in the catalyst, both initially present in the sample (Figure 9b) and formed under the action of hydrogen at 250 °C (Figure 10). As the temperature rises to 350 °C the a.b. intensity of methane increases significantly as a result of a growing amount of reduced nickel in a given temperature range (Figure 10). But later on, the FTIR spectra profile of the gas phase (Figure 12) practically does not change either in time or with increasing temperature. It indicates that the activation of the Ni-Al_2_O_3_-SSC catalyst is already complete at 350 °C, while reduction of industrial NIAP-07-01 catalyst only begins at this temperature (Figure 13). However, the complete activation of the NIAP-07-01 catalyst is not achieved at 350 °C, since the absorption band intensity of methane increases with further growth temperature as shown in Figure 13a. This effect cannot be explained only by the kinetic features of CO_2_ methanation over industrial NIAP-07-01 catalyst at different temperatures. However, a more likely reason is the gradual reduction of nickel, which is part of the nickel–aluminum spinel (Figure 9a).

Thus, the combustion of a solid-state mixture γ-Al_2_O_3_ and hexakis-(imidazole) nickel (II) nitrate complex makes it possible to synthesize a nickel–alumina catalyst for CO_2_ methanation with a lower activation temperature than for the industrial NIAP-07-01 catalyst. Herewith the active component of the NIAP-07-01 catalyst is reduced for 1 h at 350 °C, and for the Ni-Al_2_O_3_-SSC catalyst, a reduction time is only 5 min, even in the presence of CO_2_.

#### 3.3.2. Catalyst Activity in CO_2_ Methanation

**Figure 14:**
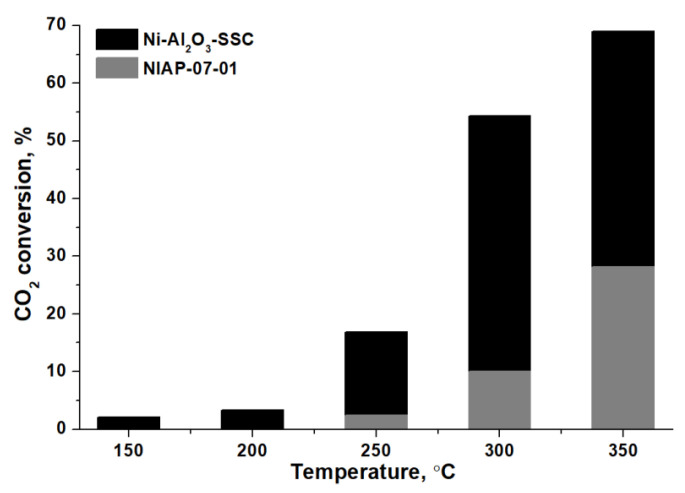
CO_2_ conversion over Ni-Al_2_O_3_-SSC and NIAP-07-01 catalysts.

CO_2_ methanation of over Ni-Al_2_O_3_-SSC and NIAP-07-01 catalysts was studied at different temperatures. The activation temperature was 450 °C to increase the percentage of nickel reduction in the industrial NIAP-07-01 catalyst (Figure 13a). Comparing the CO_2_ concentration at the inlet and outlet of the reactor (Figure 14), it was found that the Ni-Al_2_O_3_-SSC catalyst prepared by the SSC method is able to catalyze CO_2_ methanation already at 150 °C. This catalyst provides a CO_2_ conversion of about 17% without the formation of CO at 250 °C.

The industrial NIAP-07-01 catalyst starts exhibiting low activity only at 250 °C (Figure 14), like most nickel–alumina catalysts for CO_2_ methanation, reduced by pure hydrogen at 450–700 °C (Table 4). It should be noted that low amount of carbon monoxide was contained in the gaseous products of CO_2_ methanation over industrial NIAP-07-01 catalyst (Figure S6).

Therefore, the combustion of a solid-state mixture of γ-Al_2_O_3_ and hexakis-(imidazole) nickel (II) nitrate complex allows synthesizing a nickel–alumina catalyst having a high activity and selectivity in low-temperature CO_2_ methanation as compared to the commercial NIAP-07-01 catalyst. Moreover, the activity of the Ni-Al_2_O_3_-SSC catalyst is comparable to the activity of similar catalysts (Table 4) prepared by the sol-gel method [40] and by the solution combustion synthesis [34], which provide CO_2_ conversion of 17 and 44% at 250 °C, respectively. However, these values of CO_2_ conversion were obtained for nickel–aluminum catalysts with a nickel content of 20 wt%, when a reaction mixture of hydrogen and carbon dioxide (without inert gas) was fed into the reactor at a rate of 5.2 times less than in our study. This is an important point since for a catalyst prepared by the volumetric combustion method it was found that the CO_2_ conversion was decreased by 10% when the reagents feed rate is doubled.

## 4. Conclusions

In this work, a new approach to the solvent-free synthesis of CO_2_ methanation catalysts has been considered. A hexakis-(imidazole) nickel (II) nitrate complex was used as a precursor for the active component of the catalyst. This complex was prepared first using the dry-melt synthesis technique. The formation of the complex was confirmed by the results of elemental analysis, as well as XRD, ATR FTIR spectroscopy, and thermal analysis data.

The nickel–alumina catalyst for CO_2_ methanation was prepared by solid-state combustion technique without solvent. By XRD, HR TEM, and XPS methods it was shown that a nickel oxide is formed from the complex and is anchored on the surface of γ-Al_2_O_3_ particles under conditions of short-time high-temperature heating to 300 °C during combustion. In this case, a small amount of nickel is reduced to a metallic state. Nevertheless, the nickel–aluminum spinel formation cannot be prevented, but its content in the Ni-Al_2_O_3_-SSC catalyst is several times less than in the industrial NIAP-07-01 catalyst.

It should be noted that the localization of nickel oxide on the surface of the Ni-Al_2_O_3_-SSC catalyst ensures the formation of an active component already at 250 °C in the presence of CO_2_. The complete activation of this catalyst is achieved within 5 min at 350 °C. In contrast to the Ni-Al_2_O_3_-SSC catalyst, the active component of the industrial NIAP-07-01 catalyst only begins to form at this temperature. As the temperature rises to 450 °C the catalyst activity increases apparently due to the gradual reduction of nickel in nickel–aluminum spinel, which is predominant in industrial NIAP-07-01 catalyst.

For Ni-Al_2_O_3_-SSC and NIAP-07-01 catalysts activated in a reaction mixture (H_2_:CO_2_:Ar = 16:4:80) at 450 °C, the temperature dependence of their activity in CO_2_ methanation was obtained. According to the obtained results, the Ni-Al_2_O_3_-SSC catalyst provided a CO_2_ conversion of 17% at 250 °C without the formation of monoxide. This is comparable to the best similar catalysts prepared by the solution combustion synthesis. It has been demonstrated that the industrial NIAP-07-01 catalyst converts only 2% of CO_2_ at 250 °C, like most of the catalysts described in the literature (Table 4).

As a result of the research performed, two practically significant conclusions were drawn. First, the Ni-Al_2_O_3_-SSC catalyst preparation is simplified due to the absence of a solvent and long-time high-temperature calcination to form nickel oxide. Second, the Ni-Al_2_O_3_-SSC catalyst is activated in a reaction mixture within CO_2_ at 350 °C for 5 min, so no pure hydrogen supply is required in the nickel reduction.

## Figures and Tables

**Table 1:** Calculated and found values of nickel and aluminum content in Ni-Al_2_O_3_-SSC and NIAP-07-01 catalysts.

Catalyst	Metal Content, wt%		In Terms of Weight Per cent Oxides, wt%		Molar Ratio Ni/Al
	Al	Ni	Al_2_O_3_	NiO	
Ni-Al_2_O_3_-SSC					
*calculated*	42.4	15.7	80	20	0.17
*found*	29.8	16.4	56.4	20.8	0.25
NIAP-07-01					
*found*	23.4	24.7	44.1	31.3	0.48

**Table 2:** The phase composition of Ni-Al_2_O_3_-SSC and NIAP-07-01 catalysts.

Sample	Phase	Lattice Parameter of γ-Al_2_O_3_, Å	PDF Card
initial Al_2_O_3_	γ-Al_2_O_3_	7.918	10-425
Ni-Al_2_O_3_-SSC	γ-Al_2_O_3_	7.921	10-425
	NiO		47-1049
NIAP-07-01	γ-Al_2_O_3_	7.931	10-425
	NiO		47-1049
	Graphite		41-1487

**Table 3:** Texture characteristics of Ni-Al_2_O_3_-SSC and NIAP-07-01 catalysts.

Catalyst	Surface Area, m^2^·g^−1^	Pore Volume, m^3^·g^−1^	Average Pore Diameter, nm
Ni-Al_2_O_3_-SSC	157	0.35	8.8
NIAP-07-01	150	0.35	9.2

**Table 4:** Comparison of nickel–alumina catalysts for CO_2_ methanation.

Catalyst Preparation Method	Thermal Treatment Conditions	Reduction Conditions	Reaction Conditions	Ni Content, wt%	Temperature, °C	CO_2_ Conversion *, %	Reference
SSC	-	H_2_:CO_2_:Ar = 16:4:80; 100 mL·min^−1^;350 °C; 20 min	GHSV—19,000 mL·g_cat_^−1^·h^−1^ H_2_:CO_2_:Ar = 16:4:80 H_2_/CO_2_ = 4	16.4	150	2	This work
					200	3	
					250	17	
					300	54	
					350	69	
NIAP-07-01				24.7	150	0	
					200	0	
					250	2	
					300	9	
					350	26	
Ultrasound assisted co-precipitated	700 °C, 4 h	Pure H_2_; 25 mL·min^−1^; 600 °C; 2 h	GHSV—9000 mL·g_cat_^−1^·h^−1^ H2:CO_2_ = 3.5	15	250	0	[43]
					300	2	
					350	18	
				20	200	0	
					250	6	
					300	58	
					350	70	
Wet impregnation	700 °C, 5 h	20%H_2_ in helium; 700 °C; 1 h	GHSV—52,300 h^−1^H_2_:CO_2_ = 5 without any dilution; m_cat_ = 44 mg	16	250	1	[62]
					300	6	
					400	50	
					500	75	
				39	250	2	
					300	8	
					400	78	
					500	81	
Wet impregnation in presence of citric acid	400 °C, 2 h	Pure H_2_; 80 mL·min^−1^; 600 °C; 2 h	GHSV—30,000 mL·g_cat_^−1^·h^−1^ H_2_:CO_2_:Ar = 61.6:15.4:23 H_2_:CO_2_ = 3.99	10	200	2	[17]
					240	8	
					280	39	
					320	81	
				20	200	3	
					240	19	
					280	67	
					320	90	
Wetness incipient impregnation	500 °C, 4 h	20% H_2_ in helium 300 mL·min^−1^ 500 °C 1 h	GHSV—10,000 h^−1^ H_2_:CO_2_:He = 5:1:1.5 H_2_:CO_2_—5	16	200	1	[37]
					250	5	
					300	21	
					350	65	
				20	200	1	
					250	7	
					300	32	
					350	71	
Wet impregnation	450 °C, 2 h	Pure H_2_; 25 mL·min^−1^; 450 °C; 2 h	GHSV—9000 mL·g_cat_^−1^·h^−1^ H_2_:CO_2_ = 3.5	15	200	4	[64]
					250	10	
					300	47	
					350	70	
				20	200	8	
					250	42	
					300	74	
					350	78	
Wetness incipient impregnation	800 °C, 2 h	Pure H_2_ stream; 500 °C; 2 h	GHSV—60,000 mL·g_cat_^−1^·h^−1^ H_2_:CO_2_ = 4	15	250	1	[29]
					300	52	
					350	77	
Solution combustion synthesis	400 °C, 4 h	Pure H_2_ 30 mL·min^−1^; 400 °C; 2 h	GHSV—3600 mL·g_cat_^−1^·h^−1^ H_2_:CO_2_ = 4	20	250	44	[34]
					300	84	
					350	85	
Solution combustion synthesis	600 °C, 2 h	50% H_2_ in N_2_; 40 mL·min^−1^; 600 °C; 1 h	GHSV—10,000 h^−1^ H_2_:CO_2_:CO:N_2_ = 39:6.1:4.9:50	15	300	0	[45]
					350	29	
Sulfactant-free sol-gel	700 °C, 3 h	Pure H_2_ 25 mL·min^−1^; 600 °C; 2 h	GHSV—9000 mL·g_cat_^−1^·h^−1^ H_2_:CO_2_ = 3.5 without any dilution	15	200	1	[40]
					250	14	
					300	61	
					350	72	
				20	200	2	
					250	17	
					300	65	
					350	72	
Wetness incipient impregnation	600 °C, 4 h	Pure H_2_ 60 mL·min^−1^; 600 °C; 1 h	GHSV—16,000 h^−1^ H_2_:CO_2_ = 4 in N_2_	15	200	3	[65]
					250	10	
					300	28	
					350	48	

*—determined from CO_2_ conversion vs. temperature plots. The definition error is ±2%.

## Data Availability

Data is contained within the article or supplementary material.

## Supplementary Materials

The following are available online at https://www.mdpi.com/article/10.3390/ma14226789/s1, Figure S1: Reaction scheme for CO_2_ methanation over nickel catalyst, Table S1: Characterization of organometallic precursor of nickel-alumina catalyst, Figure S2: XRD pattern of hexakis-(imidazole) nickel (II) nitrate complex prepared by solvent-free dry-melt synthesis, Figure S3: FTIR spectra of gases released during heating of (a) Ni-Al_2_O_3_-SSC and (b) NIAP-07-01 catalysts (argon, 10 °C min^−1^),Table S2: Surface composition of the nickel-alumina catalysts as determined by XPS analysis, Figure S4: Survey XPS spectra of NIAP-07-01 and Ni-Al_2_O_3_-SSC catalysts, Figure S5: Low-temperature nitrogen adsorption/desorption isotherms for (a) NIAP-07-01 and (b) Ni-Al_2_O_3_-SSC catalysts, Figure S6: Effect of temperature on composition of gas at the reactor outlet during CO_2_ methanation over Ni-Al_2_O_3_-SSC and NIAP-07-01 catalysts.
