# Supplementary material for: CO2 Methanation: Nickel–Alumina Catalyst Prepared by Solid-State Combustion
Source: Materials (Basel). 2021 Nov 10;14(22):6789. doi: 10.3390/ma14226789 (PMC8623454; doi:10.3390/ma14226789)
Supplement: Supplementary file 1 [file materials-14-06789-s001.zip › materials-1419336-supplementary.pdf]

SUPPORTING INFORMATION

CO_2 Methanation: Nickel-Alumina Catalyst Prepared by Solid-State Combustion

Olga Netskina*, Svetlana Mucha, Janna Veselovskaya, Vasily Bolotov, Oxana Komova, Arkady Ishchenko, Olga Bulavchenko, Igor Prosvirin, Alena Pochtar and Vladimir Rogov

Boreskov Institute of Catalysis SB RAS, Pr. Akademika Lavrentieva 5, Novosibirsk, 630090, Russia

* Correspondence: netskina@catalysis.ru (O.V.N.); Tel.: +7-383-330-74-58

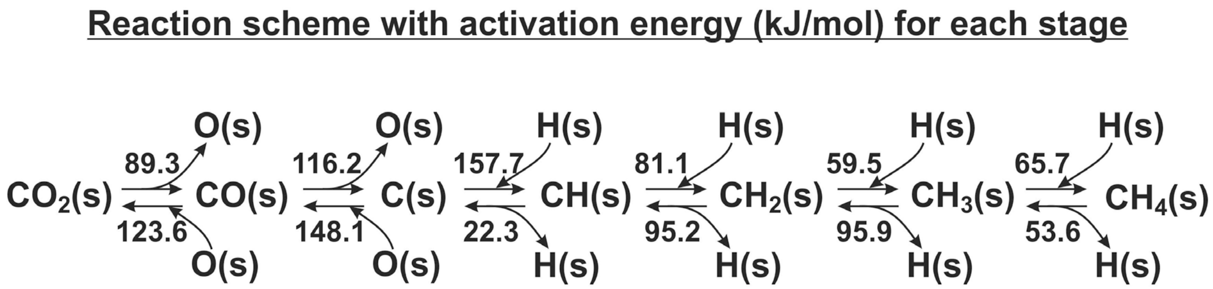

Figure S1. Reaction scheme for CO_2 methanation over nickel catalyst [Schmider, D., Maier, L., Deutschmann, O. Reaction kinetics of CO and CO_2 methanation over nickel. *Ind. Eng. Chem. Res.* **2021**, *60*, 5792–5805].

Table S1. Characterization of organometallic precursor of nickel-alumina catalyst.

Calculated composition of complex and its molar mass	Content, wt%		Oxygen balance, %
	calculated	found	
$Ni(C_3H_4N_2)_6(NO_3)_2$<br>591.2 g·mol^{-1}	Ni – 9.93<br>C – 36.57<br>H – 4.09<br>N – 33.17	Ni – 9.3<br>C –36.86<br>H – 4.55<br>N –33.88	-116.4

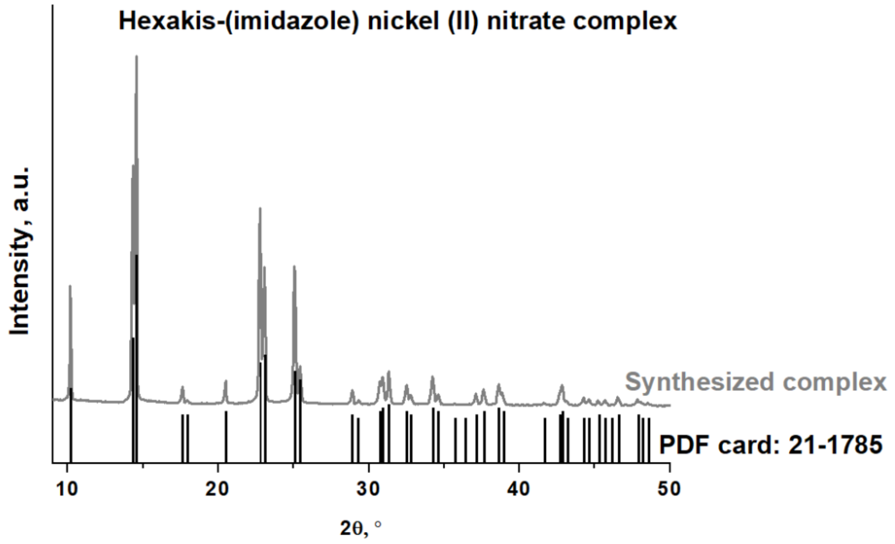

Figure S2. XRD pattern of hexakis-(imidazole) nickel (II) nitrate complex prepared by solvent-free dry-melt synthesis.

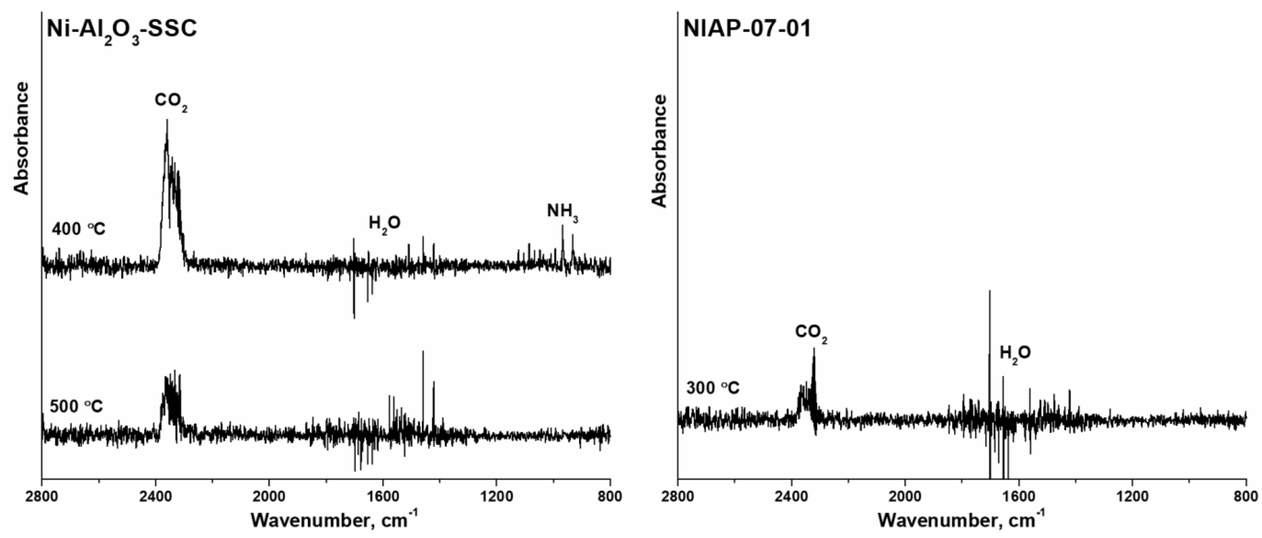

(**a**) (**b**)

Figure S3. FTIR spectra of gases released during heating of (**a**) Ni-Al_2O_3-SSC and (**b**) NIAP-07-01 catalysts (argon, 10 °C·min^{-1}).

Table S2. Surface composition of the nickel-alumina catalysts as determined by XPS analysis.

Catalyst	Content, at%					Atom ratio		
	Al	Ni	O	C	N	Ni/Al	$NiAl_2O_4$/NiO	Ni^0/$NiAl_2O_4$
NIAP-07-01	24.2	9.2	44.9	21.1	0.5	0.38	4.76	-
Ni-Al_2O_3-SSC	25.2	12.8	43.0	17.4	1.6	0.51	1.54	0.04

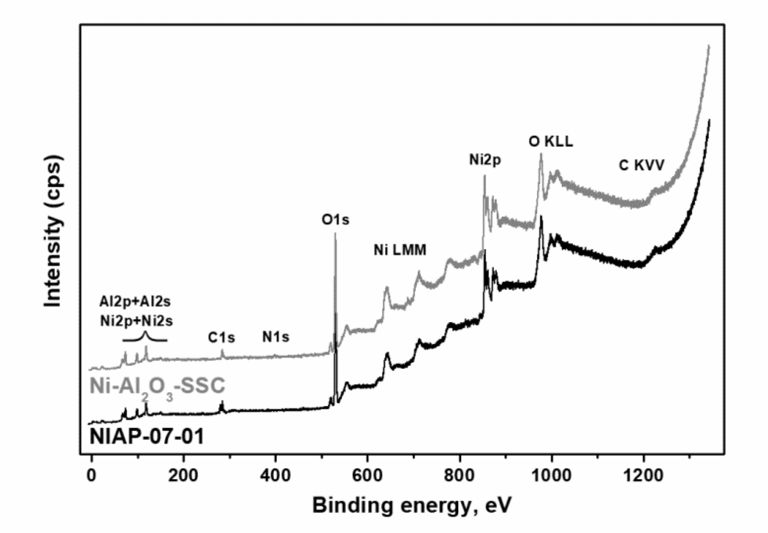

Figure S4. Survey XPS spectra of NIAP-07-01 and Ni-Al_2O_3-SSC catalysts.

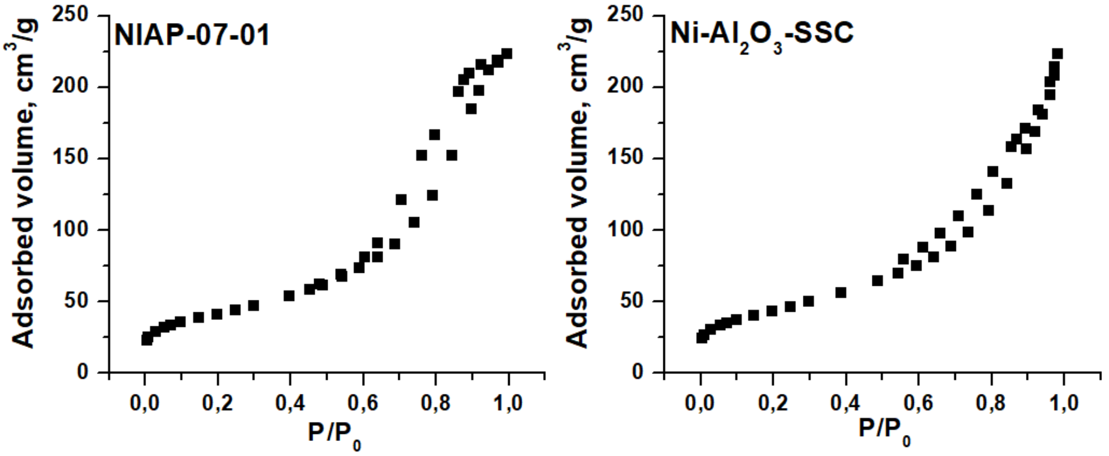

Figure S5. Low-temperature nitrogen adsorption/desorption isotherms for (**a**) NIAP-07-01 and (**b**) Ni-Al_2O_3-SSC catalysts.

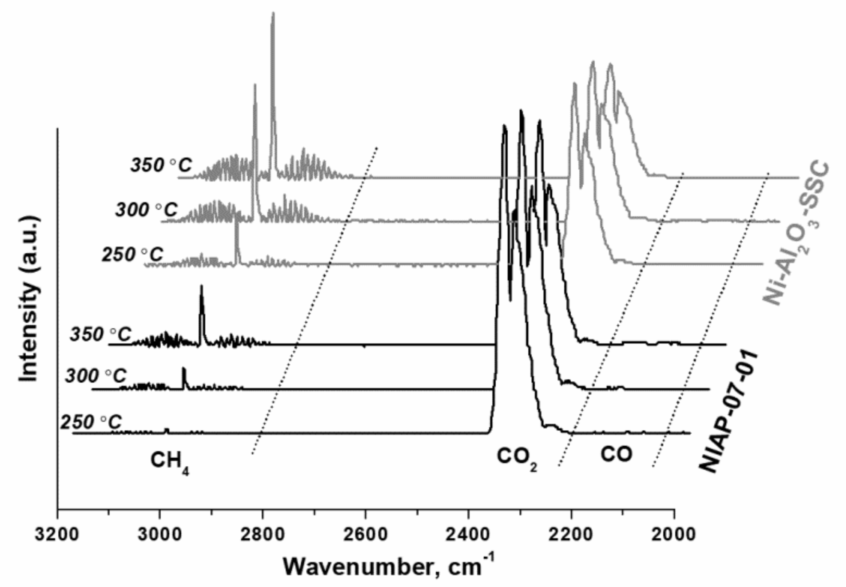

Figure S6. Effect of temperature on composition of gas at the reactor outlet during CO_2 methanation over Ni-Al2O3-SSC and NIAP-07-01 catalysts.
